# Gnathostomosis, an emerging foodborne zoonotic disease in Acapulco, Mexico.

**DOI:** 10.3201/eid0502.990211

**Published:** 1999

**Authors:** N Rojas-Molina, S Pedraza-Sanchez, B Torres-Bibiano, H Meza-Martinez, A Escobar-Gutierrez

**Affiliations:** Hospital Regional Vicente Guereo, Institute Mexicano del Seguro Social,Acapulco, Guerrero, México.

## Abstract

Between 1993 and 1997, 98 gnathostomosis cases were clinically identified in Acapulco, Mexico. Intermittent cutaneous migratory swellings were the commonest manifestation. Larvae were identified in 26 cases, while in 72, final diagnosis was made on the basis of epidemiologic data, food habits, and positive enzyme-linked immunosorbent assay and Western blot results.


### Gnathostomosis, an Emerging Foodborne Zoonotic Disease in Acapulco, Mexico

**Norma Rojas-Molina,* Sigifredo Pedraza-Sanchez,† Balfre Torres-Bibiano,* Hector Meza-Martinez,* and Alejandro Escobar-Gutierrez†**

*Hospital Regional "Vicente Guerrero," Instituto Mexicano del Seguro Social, Acapulco, Guerrero, México; and †Secretaría de Salud, México, DF, México

Between 1993 and 1997, 98 gnathostomosis cases were clinically identified in Acapulco, Mexico. Intermittent cutaneous migratory swellings were the commonest manifestation. Larvae were identified in 26 cases, while in 72, final diagnosis was made on the basis of epidemiologic data, food habits, and positive enzyme-linked immunosorbent assay and Western blot results.

Gnathostomosis is a foodborne zoonotic disease caused by several species of nematode *Gnathostoma*. The life cycle of this parasite is as follows: Adult parasites of *G. spinigerum* are found in the stomach of mammals (e.g., dogs and cats). feces containing ova reach the water (i.e., when domestic parasitized animals live at the shore of a lagoon). Free-swimming first-stage larvae are formed, which are ingested by the minute copepod crustacean cyclops, and become second-stage larvae. Freshwater fish eating cyclops are the second intermediate host. Larvae develop to the third state (L3) in the fish muscles. Consumption of this fish by cats, dogs, or other mammals results in development of adults in the gut, closing the cycle. Humans acquire the infection by consuming raw or undercooked freshwater fish. When a larva is ingested by a human host, no further development occurs, but the larva migrates through subcutaneous tissue and internal organs where it produces migratory swelling in the skin and other symptoms depending on the site or organ affected. In most cases, symptoms are not serious; however, if the parasite migrates to vital organs of the host, it can cause severe illness or even death (1,2).

Address for correspondence: Alejandro Escobar-Gutierrez, Departamento de Investigaciones Inmunológicas, Instituto Nacional de Diagnóstico y Referencia Epidemiológicos, SSA, Carpio 470, México, DF, 11340, México; fax 525-341-3264; e-mail: indre@cenids.ssa.gob.mx.

With the highest prevalence in Southeast Asia, gnathostomosis is now an emerging public health problem in Peru, Ecuador and, since 1970, in Mexico (3). In the 1970s and 1980s a few cases were reported in towns around Presidente Miguel Aleman Dam and Papaloapan River basin in the Gulf of Mexico (4). The parasites may have spread when new dams built on rivers of the Pacific Ocean coast were unnoticeably seeded with infected fish. Until now, *G. spinigerum* has been the only species of *Gnathostoma* identified in Mexico (5).

Acapulco (estimated population 890,000), a resort on the southern Pacific coast, is the major Mexican city where gnathostomosis cases have been reported (5). Between December 1, 1993, and July 31, 1997, 98 patients with symptoms compatible with gnathostomosis were identified at the outpatient clinics of the Dermatology and Allergy Services, Hospital Regional "Vicente Guerrero", IMSS. Because the hospital gives medical attention to approximately 45% of the permanent residents of the city, the number of gnathostomosis cases identified can be considered representative prevalence in the local population.

A standardized questionnaire was administered to each patient, requesting demographic data, medical history, clinical features, age, occupation, years of residence at the city, and food habits. Baseline and diagnostic investigations were made after obtaining a written consent. Gender and age distribution were as follows: 50 were male and 48 female and all but two (5 and 10 years) were 20 to 45 years of age (mean age 36 years). Blood samples were obtained for full blood and eosinophil counts. Serum total immunoglobulin E (IgE) levels were measured by a commercial enzyme-linked immunosorbent assay (ELISA) (Pharmacia Diagnostics, Uppsala, Sweden), and the results were expressed as international units (IU)/mL. Biopsies were taken from the progressing eruption and were examined for worms. Commonest symptoms were intermittent episodes of localized migratory skin swelling, with edema of variable size, slightly reddish, feverish at times, accompanied by stabbing pain, burning sensation and pruritus. Initial edema generally appeared on the abdomen. Recurring edema developed randomly, mainly in the upper and lower extremities, gluteus, thorax, and face. The duration of edema varied from 1 day to approximately 2 weeks. Only two patients reported visceral manifestations, mainly epigastrial pain and nausea, lasting approximately2 days. Medium value for blood eosinophils was 12%, and serum IgE was 302.6 Ul/mL (normal 10 to 180 Ul/mL). Although identification of larvae by a biopsy is recommended for definitive diagnosis, such identification was possible in only 16 cases, including three in which the worms were easily excised after their outward migration to the dermis as a consequence of treatment with albendazole as has been recommended (6).

To assess the effectiveness of serologic tests for diagnosis, a representative sample of patients were selected for antibody screening by ELISA (7) and Western blot confirmation (8). Antigens used were two batches of larval total extract. The first one was prepared with worms recovered from patient's biopsies or from muscles of freshwater fishes (*Dormilatum latrifons*) from laguna Tres Palos (a large freshwater lagoon 20 km southeast Acapulco). The second batch was obtained from *G. spinigerum* freeze-dried specimens donated by Dr. Wichit Rojekittkhum (Faculty of Tropical Medicine, Mahidol University, Bangkok, Thailand). For ELISA, protein concentration of the antigen was adjusted to 10 g/ml, and sera with $OD_{420}$ values ≥ the $OD_{420}$ average obtained in control sera plus four standard deviations were considered positive. ELISA-positive results were found in four of five parasitologically confirmed cases and 19 of 22 biopsy-negative patients. Immunoblots with sera from ELISA-positive patients showed two strong bands (30 and 38 kDa) and two weak bands (35 and 43 kDa) not observed in sera from the control group. No differences between the two batches of antigens were detected by ELISA or by immunoblotting. However, by ELISA, higher $OD_{420}$ values were seen when *G. spinigerum* lyophilized antigen was used. Lyophilization may make the antigen better for binding to the ELISA pates. No correlation was found with age, time of onset, or parasitologic treatment in patients with negative serologic results.

Although characteristic clinical manifestations could be very indicative of gnathostomosis, up to now confirmation through identification of larvae has been mandatory; nevertheless, larva identification is rare, and a combination of such factors as permanent residence or recent visit to a disease-endemic area, history of eating raw fish, exclusion of other diseases, and results of serologic tests can be taken into account in establishing a definitive diagnosis.

Inability to interrupt the parasite's life cycle and lack of effective medical treatment (2) make preventive measures critical in controlling the disease. Therefore, travelers to disease-endemic areas must be warned of the possibility of acquiring gnathostomosis and be instructed to avoid ingesting any form of raw fish. To protect the general population of disease-endemic areas, public campaigns should be implemented and encouraged against eating or selling raw or poorly cooked freshwater fish, especially in the form of sushimi or "ceviche" (a spicy lime-marinated fish salad of South American origin now very popular in Mexico).

Dr. Rojas-Molina is an allergologist at Service of Allergy, Hospital Regional "Vicente Guerrero," Instituto Mexicano del Seguro Social, Acapulco, Guerrero, México.

### References

1. Rusnak JM, Lucey DR. Clinical gnathostomiasis: case report and review of the English-language literature. Clin Infec Dis 1993;16:33-50.
2. Yoshimura K, 1998. *Angiostrongylus* (*Parastrongylus*) and less common nematodes. In: Cox EG, Kreier JP, Wakelin D, editors. Topley & Wilson's microbiology and microbial infections. 9th ed. Vol 5. London: Arnold; 1998. p. 635-59.

3. Pelaez D, Perez-Reyes R. Gnatostomiasis humana en America. Rev Latinoam Microbiol 1970;12:83-91.
4. Martínez-Cruz JM, Bravo-Zamudio R, Aranda-Patraca A, Martínez-Marañón R. La gnatostomiasis en México. Salud Pública Mex 1989;31:541-9.
5. Ogata K, Nawa Y, Akahane H, Díaz Camacho SP, Lamothe-Argumedo R, Cruz-Reyes A. Short report: gnathostomiasis in Mexico. Amer J Trop Med Hyg 1998;58:316-8.
6. Suntharasamai P, Riganti M, Chittamas S, Desakorn V. Albendazole stimulates outward migration of *Gnathostoma spinigerum* to the dermis in man. Southeast Asian J Trop Med Public Health 1992;23:716-22.
7. Dharmkrong-at A, Migasena S, Suntharasamai P, Bunnag D, Priwan R, Sirisinha S. Enzyme-linked immunosorbent assay for detection of antibody to *Gnathostoma* antigen in patients with intermittent cutaneous migratory swelling. J Clin Microbiol 1986;23:847-51.
8. Towbin H, Straehelin T, Gordon J. Electrophoresis transfer of proteins from polyacrylamide gels to nitrocellulose sheets: procedure and some applications. Proc Natl Acad Sci U S A 1979;76:4350-4.
